# *Aspergillus oryzae* PrtR alters transcription of individual peptidase genes in response to the growth environment

**DOI:** 10.1007/s00253-023-12833-5

**Published:** 2024-01-10

**Authors:** Rika Numazawa, Yukako Tanaka, Sawako Nishioka, Ryotaro Tsuji, Hiroshi Maeda, Mizuki Tanaka, Michio Takeuchi, Youhei Yamagata

**Affiliations:** 1 Department of Applied Biological Science, United Graduate School of Agricultural Science, Tokyo University of Agriculture and Technology, 3-5-8 Saiwai-Cho, Fuchu, Tokyo, 1838509 Japan; 2 Department of Applied Biological Chemistry, Graduate School of Agriculture, Tokyo University of Agriculture and Technology, 3-5-8 Saiwai-Cho, Fuchu, Tokyo, 1838509 Japan

**Keywords:** *Aspergillus oryzae*, Transcriptional factor, PrtR, Extracellular peptidase

## Abstract

**Abstract:**

*Aspergillus oryzae* PrtR is an ortholog of the transcription factor PrtT, which positively regulates the transcription of extracellular peptidase genes in *Aspergillus niger* and *Aspergillus fumigatus*. To identify the genes under the control of PrtR and elucidate its regulatory mechanism in *A. oryzae*, *prtR* gene disruption mutants were generated. The control strain clearly showed a halo on media containing skim milk as the nitrogen source, whereas the ΔprtR strain formed a smaller halo. Measurement of acid peptidase activity revealed that approximately 84% of acidic endopeptidase and 86% of carboxypeptidase activities are positively regulated by PrtR. As the transcription of the *prtR* gene varied depending on culture conditions, especially with or without a protein substrate, it was considered that its transcription would be regulated in response to a nitrogen source. In addition, contrary to previous expectations, PrtR was found to act both in promoting and repressing the transcription of extracellular peptidase genes. The mode of regulation varied from gene to gene. Some genes were regulated in the same manner in both liquid and solid cultures, whereas others were regulated in different ways depending on the culture conditions. Furthermore, PrtR has been suggested to regulate the transcription of peptidase genes that are closely associated with other transcription factors.

**Key points:**

*• Almost all peptidase genes in Aspergillus oryzae are positively regulated by PrtR*

*• However, several genes are regulated negatively by PrtR*

*• PrtR optimizes transcription of peptidase genes in response to culture conditions*

**Supplementary Information:**

The online version contains supplementary material available at 10.1007/s00253-023-12833-5.

## Introduction

*Aspergillus oryzae* is a filamentous fungus that is used as a source of enzymes for producing traditional Japanese fermented foods (Machida et al. 2008). Furthermore, *A. oryzae* is considered as a microorganism that can be safely used for food production and is listed in the US Food and Drug Administration’s generally recognized as safe (Taylor and Richardson 1979). The fungus has a strong ability to secrete hydrolytic enzymes, mainly amylases and peptidases, and a superior protein secretion ability. Therefore, this fungus is used as a host for the production of native and heterologous enzymes in the enzyme industry (Papagianni 2004). This fungus is also expected to be used in the production of pharmaceutical raw materials, and the demand for these products is increasing (Yoon et al. 2011; Huynh et al. 2020). Despite the industrial value of *A. oryzae*, the genetics and molecular biology of the fungi could not be studied, because the sexual generation required for classical genetic analysis has not been identified. In 2001, expressed sequence tag (EST) analysis of *A. oryzae* RIB40 was conducted with the development of molecular biological experimental methods (Akao et al. 2007). Whole-genome sequence analysis was completed in 2005 (Machida et al. 2005), and 5 years later, Wang et al. (2010) published the results of transcriptome analysis using large-scale RNA-seq. These results have accelerated the molecular biological study of the fungus. Genome analysis has revealed that *A. oryzae* contains approximately 130 peptidase genes. Meanwhile, *Aspergillus nidulans* (Galagan et al. 2005) and *Aspergillus fumigatus* (Nierman et al. 2005), whose genome analyses were reported simultaneously with *A. oryzae*, showed 90 and 99 peptidase genes, respectively. In other words, *A. oryzae* was found to have more peptidase genes than other closely related species. Phylogenetic analysis suggested that the peptidase genes of *A. oryzae* would have evolved to have diverse homologous enzymes through gene duplication (Machida et al. 2005). In other words, it has been assumed that *A. oryzae* would have evolved with an increased number of peptidase genes to be able to degrade a variety of substrates. We have considered that *A. oryzae* might have subdivided the roles of each peptidase more than other Aspergilli. Thus, peptidases are thought to be important enzymes in characterizing *A. oryzae*. However, it is still unclear which transcription factors are involved in the transcription of each peptidase gene, the DNA sequences to which they bind, and how transcriptional induction occurs.

The control of peptidase production in *A. oryzae* has contributed to various enzyme industries. As mentioned above, *A. oryzae* is a safe filamentous fungus; therefore, peptidases derived from *A. oryzae* are used in various food-processing applications (Machida et al. 2008). Since large quantities of enzymes are often used in food processing, it is preferable that the culture supernatant be used directly, as an enzyme agent. However, as the fungus can produce a wide variety of peptidases, it is difficult to make the fungi produce only certain enzymes. Many enzyme manufacturers have been attempting to control peptidase production under culture conditions. Nonetheless, without knowing how peptidase genes are transcriptionally regulated, trial and error is the only method. Understanding how transcription is regulated will help to control peptidase production. In addition, by achieving efficient production of the required peptidases in the brewing field, fermentation foods can be produced more efficiently. Furthermore, if peptidase production can be suppressed in using *A. oryzae* as a host for heterologous protein expression, the degradation of expressed proteins can be prevented by the proteolytic enzymes produced by *A. oryzae*.

Two transcription factors, FlbC and PrtT, are involved in the production of extracellular peptidases in *Aspergillus*. FlbC was identified as a transcription factor that positively regulates the transcription of the gene encoding glucoamylase B (GlaB), which is specifically produced in solid cultures (Tanaka et al. 2016). Furthermore, FlbC has been shown to positively regulate *pepO* and the neutral peptidase gene, *nptB* (*deuA*), in solid culture (Tanaka et al. 2016). PrtT was first identified in *Aspergillus niger* by Punt et al. (2008) and is also a transcription factor belonging to the Zn(II)_2_Cys_6_ binuclear cluster family. The gene, *prtT*, is located close to the starch-degrading gene cluster. The *prtT* family has been suggested to be unique to the genera *Aspergillus* and *Penicillium* (Sharon et al. 2009). Transcriptome analysis using the *prtT* deletion and overexpression strains of *A. niger* showed transcriptional changes in 32 putative protease genes, and the binding DNA sequence was deduced as 5′-CCGHCGG-3′ (H; A/C/T) by MEME motif-based sequence analysis (Huang et al. 2020). Moreover, the transcriptional levels of several extracellular peptidase genes have been reported to be reduced in an *A. fumigatus* ΔprtT strain compared to those in the control strain (Bergmann et al. 2009). In addition, RNA-seq using an *Penicillium oxalicum* ΔprtT strain and MEME analysis/electrophoretic mobility shift assay (EMSA) using the upstream region of genes whose transcription levels were altered by *prtT* deletion showed that the binding sequence was 5′-CHGH(D)CGG-3′ (H; A/C/T, D; A/C/T) (Chen et al. 2014).

The ortholog of PrtT in *A. oryzae* has been named PrtR (AO090003001577). PrtR, like PrtT, regulates the transcription of peptidase genes, and *prtR* is found to be present close to the starch-degrading gene cluster (Gomi 2019). Protease and acid carboxypeptidase activities are increased in *prtR* overexpression strain, and the transcription level of the alkaline protease (oryzin) gene *alpA* is increased by more than fivefold (Takahashi et al. 2018). The halo formed in skim milk medium is larger in *prtR*-overexpression strain, and the transcriptional levels of the dipeptide- and tripeptide-transporter genes *potA* and *potB* are increased by the overexpression of *prtR* and decreased by the loss of *prtR* (Tanaka et al. 2021). Thus, PrtR plays a major role in the regulation of gene expression in the presence of proteins as a nitrogen source for their utilization. However, the scope of the influence of PrtR and the detailed mechanism of its transcriptional regulation remain unclear. In this study, to evaluate the role of PrtR as a regulator of peptidase genes in *A. oryzae*, we constructed a PrtR deletion mutant strain without nutrient requirements for nitrogenous substances and examined the changes in the transcription levels of the extracellular peptidase genes in liquid and solid cultures. We clarified the peptidase genes under the regulation of PrtR and further investigated the changes in the regulation mode of PrtR in liquid and solid cultures to elucidate the regulatory mechanism of PrtR.

## Material and methods

### Strains, media, and growth condition

*A. oryzae* strains, used in the present study, are listed in Table 1. The strains were grown at 30 °C in Czapek-Dox (CD) medium (Fujioka et al. 2007) at pH 5.2. According to the nutrient requirement or drug resistance, 20 mM uracil and 1.1 mg/mL 5-fluoroorotic acid or 0.1 µg/mL pyrithiamine were added. For the solid culture, wheat bran medium prepared by adding 6 mL of reverse osmosis water to 4.2 g of wheat bran (Showa Sangyo Co., Ltd., Tokyo, Japan) was used.

**Table 1:** *Aspergillus oryzae* strains used in this study

Strain	Genotypes	References
RIB40	WT	ATCC42149 (Akao et al. 2007)
ΔligD ΔpyrG	Δ*ligD*::*ptrA* Δ*pyrG*	(Kobayashi et al. 2017)
ΔligD ΔpyrG ΔprtR::[pyrG cre]	Δ*ligD*::*ptrA* Δ*pyrG* Δ*prtR*::[*pyrG cre*]	This study
ΔligD ΔpyrG ΔprtR	Δ*ligD*::*ptrA* Δ*pyrG* Δ*prtR*	This study
ΔprtR	Δ*ligD*::[*pyrG ligD*] Δ*pyrG* Δ*prtR*	This study
Control	Δ*ligD*::[*pyrG ligD*] Δ*pyrG*	This study

For growth observation, CD agar medium was used; however, 0.6% bovine serum albumin (BSA) (CD/BSA), 0.6% gelatin (CD/gelatin), 0.6% soy protein (CD/soy protein), 1% casein (CD/casein), and 1% skim milk (CD/SM (− N)) were used as a single nitrogen source, instead of NaNO_3_. CD agar medium containing 0.6% NaNO_3_ and 1% skim milk (CD/SM (+ N)) was also prepared. In addition, CD agar medium containing 2% soluble starch instead of Glc (CD/starch) was used.

*Escherichia coli* DH5α (F^−^, $\Phi$ 80d *lacZ*DM15, D(*lacZYA*-*argF*) U169, *deoR*, *recA*1, *endA*1, *hsdR*17 (r_K_^−^ m_K_^+^), *phoA*, *supE*44, λ^−^, *thi*-1, *gyrA*96, *relA*1) (Takara Bio Inc., Kusatsu, Japan) was used for plasmid construction (Chen et al. 2018).

### Construction of prtR deletion strain

The cassette for the *prtR* gene deletion was constructed as follows. Primer sets for deletion mutant construction are listed in Supplemental Table S1. A DNA fragment from 2 kb upstream to 2 kb downstream of the *prtR* open reading frame (ORF) was obtained by PCR using phosphorylated primers (prtR_5′_F and prtR_3′_R) and *A. oryzae* RIB40 genomic DNA as a template. The fragment was, then, inserted into the *Hin*cII-treated pUC119 (Takara Bio Inc., Kusatsu, Japan) vector. The constructed plasmid was named as pUC119-prtR. Next, inverse PCR was performed using primers (prtR_3′_F and prtR_5′_R) and the pUC119-prtR as templates to remove the *prtR* ORF from the pUC119-prtR. The obtained DNA fragment was ligated with the DNA fragment containing *adeA* (from *A. nidulans*) and Cre/*loxP* expression genes obtained by PCR using primers (creloxp_F and creloxp_R) and the pAAAXP_Cre (Zhang et al. 2017) as a template. The plasmid pAAAXP_Cre was kindly gifted by Dr. Gomi (Tohoku University). The resulting plasmid was named pUC119_prtRarm_AnadeA_cre. To substitute the *adeA* gene in the pUC119_prtRarm_AnadeA_cre with the *pyrG* gene, the *pyrG* gene fragment was obtained by PCR using phosphorylated primers (pyrG_Fw and pyrG_Rv) and *A. oryzae* RIB40 genomic DNA as a template. Meanwhile, inverse PCR was performed using primers (creloxp_5′_F and creloxp_3′_R) and the pUC119_prtRarm_AnadeA_cre as a template to remove the *adeA* gene region from the pUC119_prtRarm_AnadeA_cre. These two fragments were ligated, and the resulting plasmid was named pUC119_prtRarm_pyrG_cre. As a result, the 2-kb upstream region of the *prtR* ORF, the *pyrG* selectable marker, the Cre/*loxP* expression gene, and the 2-kb downstream region of *prtR* ORF were arranged in this order from the 5′ end of the cassette for recombination.

This cassette was used for transformation of the *A. oryzae* ΔligD::ptrA ΔpyrG strain (Kobayashi et al. 2017) by the protoplast-PEG method (Gomi et al. 1987), and subsequent selection for deletion of the *prtR* gene was performed on CD agar medium containing 0.1 µg/mL pyrithiamine. Loop-out of the *pyrG* gene by the Cre-*loxP* system was conducted on CDX agar medium (CD agar medium with 2% xylose as the sole carbon source), as described previously (Zhang et al. 2017). After loop-out of the *pyrG* gene, the *ligD* and *pyrG* genes were complemented using pUC119-ligD-pyrG, as described previously (Kobayashi et al. 2017).

### Preparation of genomic DNA and Southern blot analysis

Genomic DNA preparation and Southern blot analysis were performed as previously described (Kobayashi et al. 2017). The probes used for Southern blot analysis were amplified using the primers prtR_up2049_F and prtR_up967_R for the left probe and prtR_down97_F and prtR_down1045_R for the right probe to confirm the deletion of the *prtR* gene, and ligD_up_F and ligD_up_R for the left probe and ligD_down_F and ligD_down_R for the right probe of the *ligD* and *pyrG* gene complementation.

### Measurement of enzymatic activities

Twenty-five million conidia of either the control or ΔprtR strains were inoculated into 40 mL of CD-casein (1% casein was used as a single nitrogen source, instead of NaNO_3_) medium in a 120-mL baffled flask and cultured aerobically at 30 °C for 36 h. Culture supernatants were collected by filtration using Miracloth® (Merck KGaA, Darmstadt, Germany) and then centrifuged at 10,000* g* and 4 °C for 30 min. The supernatant was dialyzed against a 10 mM acetate buffer (pH 5.0) at 4 °C.

Acidic endopeptidase activity was measured as previously described (Takyu et al. 2022). In brief, 0.02 mL of enzyme solution diluted with 0.18 mL of 0.1 M sodium acetate buffer (pH3.0) was preincubated at 30 °C for 10 min, and then, 0.2 mL of 2.0% casein solution (pH3.0) preincubated at 30 °C was added to the enzyme solution. After exactly 60 min of incubation at 30 °C, 0.4 mL of 0.4 M trichloroacetic acid solution was added to stop the enzyme reaction. After leaving the mixture at 30 °C for 20 min, the mixture was centrifuged at 15,000* g* for 20 min. One hundred microliters of the supernatant was moved to a new test tube, and 0.5 mL of 0.4 M sodium carbonate solution and 0.1 mL of 0.33 M Folin & Ciocalteu’s phenol reagent were added to the supernatant. After 30 min of incubation at 30 °C, the absorbance of the mixture at 660 nm was measured using a Hitachi UV-1900 spectrophotometer (Hitachi High-Technologies Corp., Tokyo, Japan). One katal of the aspartic endopeptidase is defined as the enzyme amount, which yields the equivalent of 1.0 mol tyrosine per second at 660 nm, using casein as a substrate under the conditions described above. Inhibitory analysis was performed with 0.1 mM antipain (Peptide Institute, Inc., Osaka, Japan) or 0.1 mM pepstatin A (Peptide Institute, Inc., Osaka, Japan). Carboxypeptidase (CPase) activity was measured according to a previously reported method (Tanaka et al. 1970; Morita et al. 2009). One millimolar benzyloxycarbonyl-L-glutamyl-L-tyrosine (Z-Glu-Tyr) was used as a substrate at pH 3.5 in 50 mM sodium acetate buffer. The amount of Tyr liberated from Z-Glu-Tyr was determined as follows: 0.01 mL of enzyme solution diluted with 0.09 mL of 0.1 M sodium acetate buffer (pH 3.5) was preincubated at 30 °C for 10 min, and then, 0.1 mL of 1 mM Z-Glu-Tyr solution (pH3.5) preincubated at 30 °C was added to the enzyme solution. After exactly 60 min of incubation at 30 °C, 0.1 mL of 0.3 M NaOH was added to terminate the reaction. After leaving the mixture at 30 °C for 10 min, 0.2 mL of the mixture was moved to a new test tube, and then, 0.2 mL of 2.5% acetic acid and 2 mL of 0.5 M citrate buffer (pH 5.0) were added. Then, 1 mL of ninhydrin solution (0.83% ninhydrin, 0.17 mM KCN, dissolved in 2-methoxyethanol) was added, and the mixture was heated at 100 °C for 20 min and immediately cooled in an ice water bath. The absorbance of the mixture was measured at 570 nm using the Hitachi UV-1900 spectrophotometer. One katal of the CPase is defined as the enzyme amount, which yields the equivalent of 1 mol tyrosine per second, using Z-Glu-Tyr as a substrate under the conditions described above.

### Determination of protein concentration

Protein concentrations were measured by the Bradford method using the Bradford Protein assay kit (Bio-Rad Laboratories K. K., Tokyo, Japan) according to the supplier’s protocol (Bradford 1976).

### RNA extraction, cDNA synthesis, and quantitative RT-PCR

For liquid culture, 2 × 10^7^ conidia of the control and ΔprtR strains were inoculated into 40 mL of CD medium or CD-casein medium containing 1% casein as a solo nitrogen source without NaNO_3_ in a 120-mL baffled flask (Corning Inc., Corning, NY, USA). They were cultured aerobically at 30 °C for 24, 36, and 48 h. RNA extraction, cDNA synthesis, and quantitative RT-PCR were performed as described previously (Kobayashi et al. 2017). Primer sets used for RT-qPCR are listed in Supplemental Table S1.

For solid culture, 3 × 10^7^ conidia of the control and ΔprtR strains were inoculated into 10.2 g of wheat bran medium in Erlenmeyer flask (wide neck: Paul Marienfeld GmbH & Co. KG, Lauda-Königshofen, Germany) and cultured at 30 °C for 24, 36, 42, and 48 h. The flasks were shaken every 12 h to mix the media. RNA extraction was performed using a previously described method (Cathala et al. 1983) with some modifications. The mycelia grown on wheat bran were ground to a fine powder in liquid N_2_ using a mortar and pestle. Two and half grams of the ground mycelia were suspended in 10 mL of Sepasol®-RNA I SuperG (Nacalai Tesque Inc., Kyoto, Japan) preheated to 50 °C. Then, 4 mL of chloroform was added, and the mixture was centrifuged at 4 °C and 7,500* g* for 15 min. The aqueous layer was then subjected to phenol/chloroform extraction. RNA was precipitated using 2-propanol. To this precipitate, 2 mL of guanidine thiocyanate (GuSCN) solution [2.5 M GuSCN, 25 mM Tris–HCl (pH 7.5), 5 mM ethylenediaminetetraacetic acid; EDTA (pH 8.0)], 100 µL of 2-mercaptoethanol, and 10 mL of 4 M LiCl were added, followed by incubation at room temperature (25 °C) for 20 min and at 4 °C overnight. The mixture was centrifuged at 4 °C and 500* g* for 5 min, and 2 mL of supernatant was centrifuged at 4 °C and 11,000* g* for 90 min. The precipitate was suspended in a mixture of 500 µL of GuSCN solution, 50 µL of 2-mercaptoethanol, and 2 mL of 4 M LiCl and further centrifuged at 4 °C and 11,000* g* for 60 min. The resulting precipitate was dissolved in 5 mL of TESDS [10 mM Tris–HCl (pH 7.5), 1 mM EDTA (pH 8.0), and 0.1% sodium dodecyl sulfate]. The solution was, then, subjected to phenol/chloroform extraction and chloroform extraction. Next, the RNA was precipitated with 2-propanol and dissolved in diethylpyrocarbonate-treated water. DNase I treatment of total RNA, cDNA synthesis, and RT-PCR were performed as described previously (Kobayashi et al. 2017). The primers used for RT-qPCR are listed in Supplemental Table S1.

## Results

### Construction of a prtR deletion strain

To clarify the role of PrtR in *A. oryzae*, we constructed a deletion mutant of the *prtR* gene (Supplemental Fig. S1A). The ΔligD ΔpyrG strain was used as the host for the transformation (Kobayashi et al. 2017). We also constructed a control strain by complementing the deletion of *ligD* and *pyrG* in the *A. oryzae* ΔligD ΔpyrG strain. These strains were confirmed by Southern blotting (Supplemental Fig. S1A-S1E).

### Growth of ΔprtR in various media

**Fig. 1:**
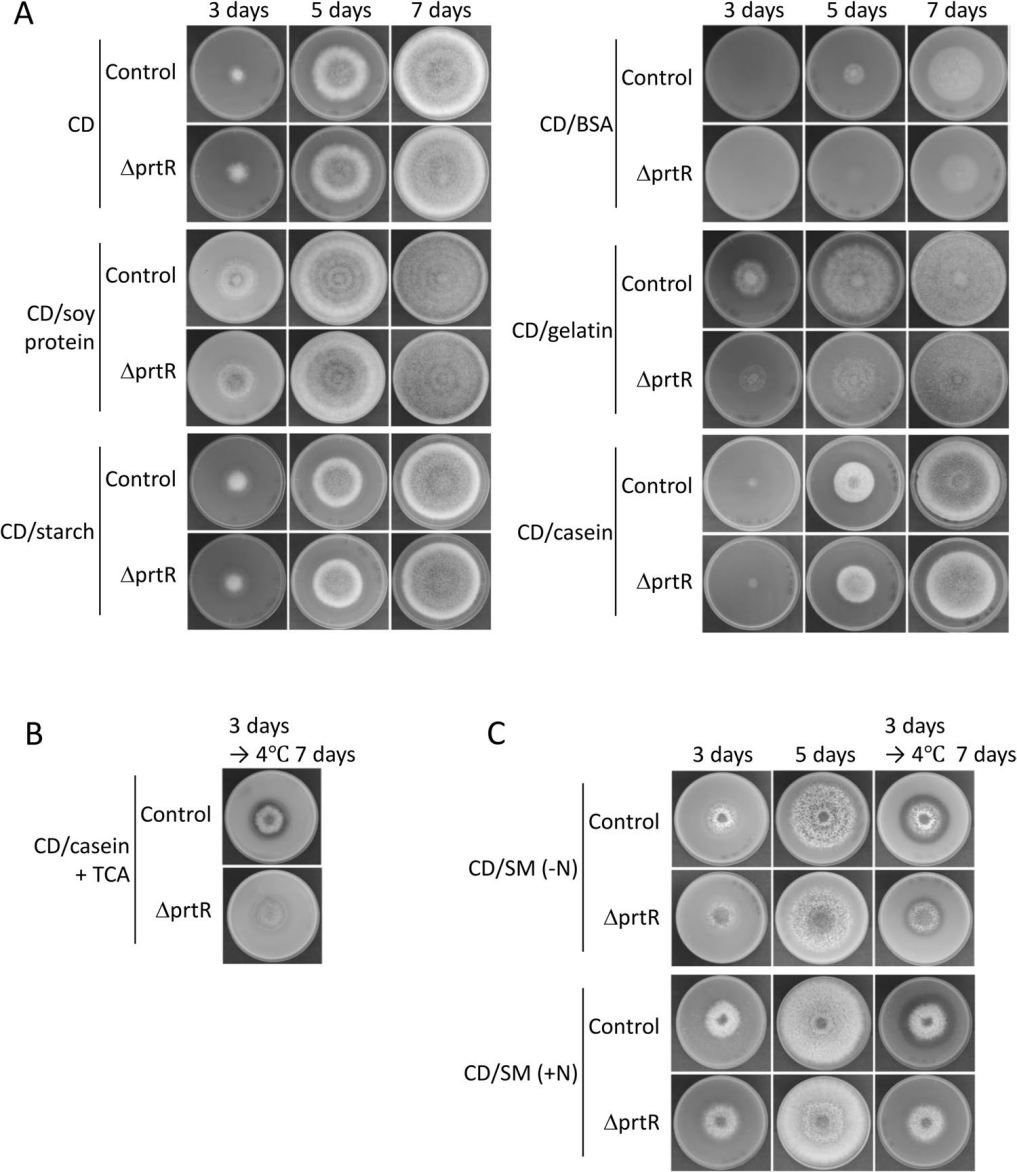
Characterization of PrtR-dependent growth. One thousand conidia of control or ΔprtR strains were inoculated onto CD agar medium containing 0.6% NaNO_3_, 0.6% BSA, 0.6% gelatin, 0.6% soy protein, 1% casein as single nitrogen source, or 2% starch as single carbon source. These were cultured at 30 °C for 3, 5, and 7 days. **A** Colony appearance. **B** Halo assay using casein. Control and ΔprtR strains were inoculated into CD/casein agar medium and cultured at 30 °C for 3 days. After 3 days, they were incubated at 4 °C for 7 days. Furthermore, 0.4 M trichloroacetic acid (TCA) was added, and the proteolytic haloes were observed. **C** Colony appearance and halo assay using skim milk. Control and ΔprtR strains were inoculated into CD/SM(-N) and CD/SM(+ N) agar medium at 30 °C for 3 and 5 days. After 3 days, they were incubated at 4 °C for 7 days and the proteolytic haloes were observed. CD/SM agar plates contained skim milk, and “ + N” contained NaNO_3_, and “ − N” plate did not

As PrtR is an ortholog of PrtT in *A. niger* and *A. fumigatus*, it is likely that PrtR also promotes the transcription of peptidase genes. We attempted to confirm whether PrtR deficiency reduced proteolytic enzyme production. Furthermore, we hypothesized that the function of PrtR could be altered by the surrounding nitrogen source. Therefore, the control and ΔprtR strains were grown on various CD media with different nitrogen sources, and their growth was compared (Fig. 1A). No significant difference was observed in growth between the control and ΔprtR strains in CD and CD/soy protein agar media containing NaNO_3_ or soy protein as a single nitrogen source. However, when BSA, gelatin, or casein was used as a single nitrogen source, the growth of ΔprtR strain was delayed compared to the control strain. Furthermore, as the *prtR* gene was originally presented close to the starch degradation cluster, glucose in CD agar medium was substituted with soluble starch to investigate whether carbon catabolite repression is involved in PrtR function. No difference in growth was observed between the control and ΔprtR strains on the CD/starch agar medium.

To confirm the secretion of proteolytic enzymes, the control and ΔprtR strains were grown on casein-containing agar medium for 3 days, following which the plates were incubated at 4 °C for 7 days. *A. oryzae* have a fast substrate mycelial elongation speed. As a result, the mycelium grows over any halo formed in a medium, and it is difficult to confirm the halo size. Therefore, the plates were placed at 4 °C to stop mycelial growth and to make halo formation easier to see. Then, 0.4 M trichloroacetic acid was added to the plates, and the halos formed (Fig. 1B). A halo was formed around the colony of the control strain, whereas no halo was observed in the ΔprtR strain. Next, to observe the relationship between the inorganic nitrogen source and PrtR in the secretory production of proteolytic enzymes, the ΔprtR strain was inoculated on both CD/SM (− N) and CD/SM (+ N) agar plates, and halos were observed as described above (Fig. 1C). Both control and ΔprtR strains formed halos on the plate without NaNO_3_ (CD/SM (− N)); however, the size of the halo was larger in the control strain. In contrast, on CD/SM (+ N) medium containing NaNO_3_, only the control strain formed a halo, and the ΔprtR strain could not.

### Peptidase production of ΔprtR

**Fig. 2:**
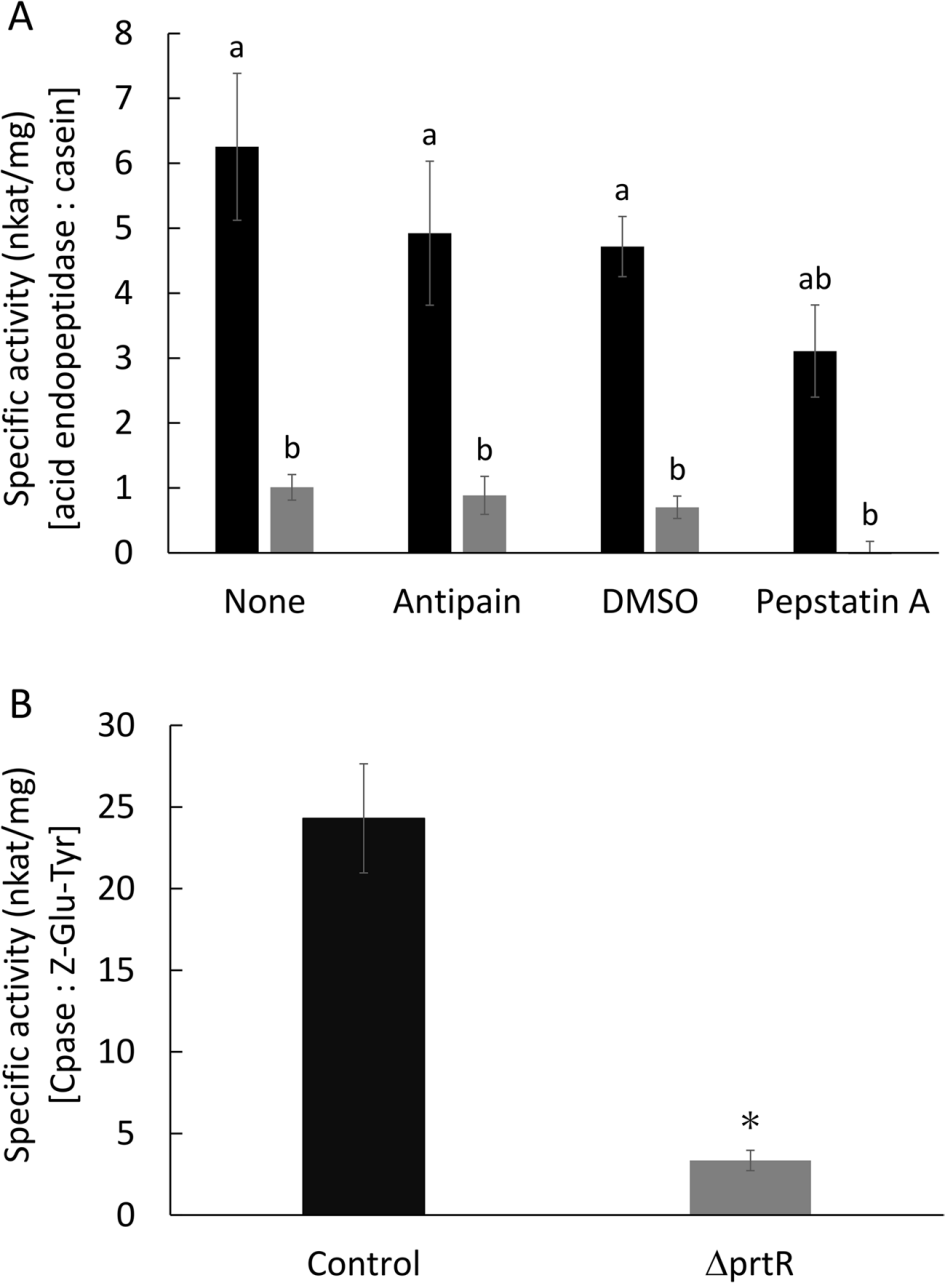
Proteolytic activity of the ΔprtR strain. Control or ΔprtR strains were grown for 36 h in CD-casein liquid medium containing 1% casein as single nitrogen source. **A** Specific activities of acid endopeptidases. Activities of the culture supernatants were measured using 2% casein (pH 3.0) as a substrate. None; no inhibitor. Antipain; cysteine and serine protease inhibitor, antipain added. Dimethyl sulfoxide (DMSO); the solvent for pepstatin A, DMSO added. Peptatin A; APase inhibitor, pepstatin A added. **B** Specific activities of acid carboxypeptidases. Activates of the culture supernatant were measured using 1 mM Z-Glu-Tyr (pH 3.5) as a substrate. The bars represent the mean of biological replicates (*n* = 3), and error bars represent the standard error. The black bars show the results of the control strain, and the gray bars show the results of the ΔprtR strain. Statistical analysis was performed using the Tukey–Kramer and *t* test method. *p < 0.05

To confirm how PrtR deletion affects peptidase activity, the activities of endopeptidases and carboxypeptidases in the culture supernatants were measured under acidic conditions. In the production of fermented foods, the environment becomes acidic in the initial stage due to lactic acid from halophilic lactic acid bacteria. The peptidases from *A. oryzae* need to degrade the ingredients of miso and soy source under such acidic condition. Therefore, acid peptidases are important for the industrial use of *A. oryzae*. The specific activities of acidic endopeptidase in the culture supernatants from the ΔprtR and control strains were 1.0 and 6.3 nkat/mg, respectively, which indicated that approximately 84% of the original activity was lost due to the deletion of PrtR. To confirm what kind of endopeptidase activities were decreased, we performed an inhibitor assay using pepstatin A and antipain. The activity of the control strain was reduced to approximately 66% and 79% of the original activity by pepstatin A and antipain, respectively (Fig. 2A). This indicated that approximately 34% of the acidic caseinolytic activity was due to aspartic endopeptidases (APases) and approximately 21% of the activity was caused by antipain-sensitive endopeptidases. In the ΔprtR strain, the activity was significantly lost by pepstatin A, and the addition of antipain hardly changed the activity. This suggested that PrtR is involved in multiple acidic endopeptidase production, such as APases and antipain-sensitive endopeptidases.

The carboxypeptidase (CPase) activities of the control strain and the ΔprtR strain were 24.3 and 3.3 nkat/mg, respectively (Fig. 2B). These results indicated that approximately 86% of the original CPase activity was lost due to the deletion of PrtR.

These results suggest that PrtR plays an important role in the production of both endopeptidases and exopeptidases.

### Transcription of peptidase genes in the ΔprtR strain cultured on liquid medium

As the proteolytic activities were reduced by PrtR deficiency, we analyzed the transcription of all genes encoding the presumed extracellular peptidases to identify the genes transcriptionally regulated by PrtR.

**Fig. 3:**
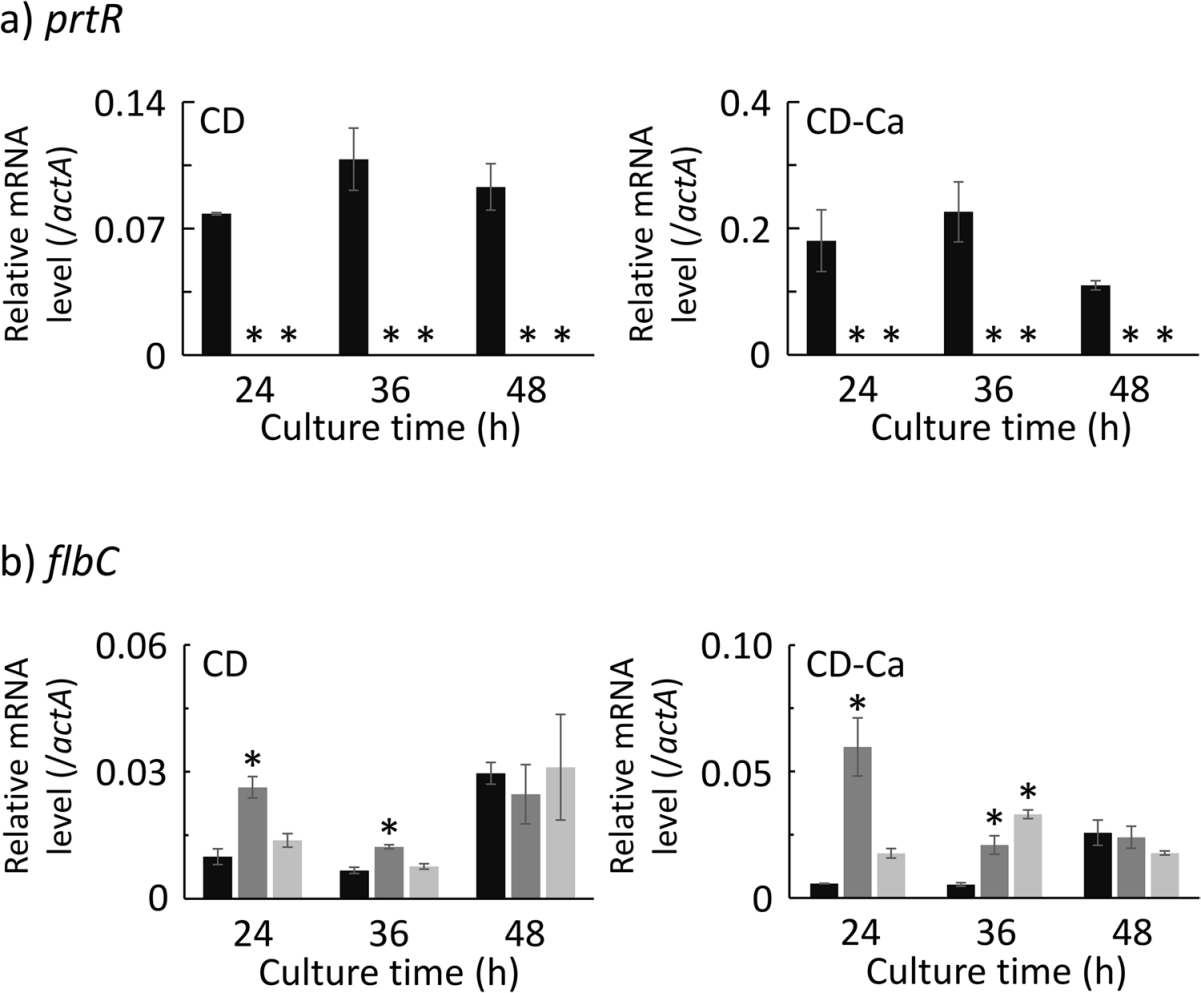
Transcriptional analysis result for transcription factor genes in the control and ΔprtR strains cultured in liquid medium. Control or ΔprtR strains were grown for 24, 36, and 48 h in CD liquid medium or CD-casein liquid medium containing 1% casein as solo nitrogen source. The *y*-axis represents the relative mRNA levels of the transcription factor gene compared to that of *actA*. The bars represent the mean of biological replicates (*n* = 3), and error bars represent the standard error. The black bars show the results of the control strain, and the two gray bars show the results of the ΔprtR strain. Statistical analysis was performed using the Dunnett method. *p < 0.05

The genes analyzed are shown in Supplemental Table S2. First, transcription of *prtR* was observed in the control strain at all time points in both media but not in the ΔprtR strain (Fig. 3). Thus, it was confirmed that the *prtR* gene was deficient. The transcriptional levels of *prtR* in CD-Ca medium at 24 and 36 h of cultivation were approximately twofold higher than those in CD medium.

**Fig. 4:**
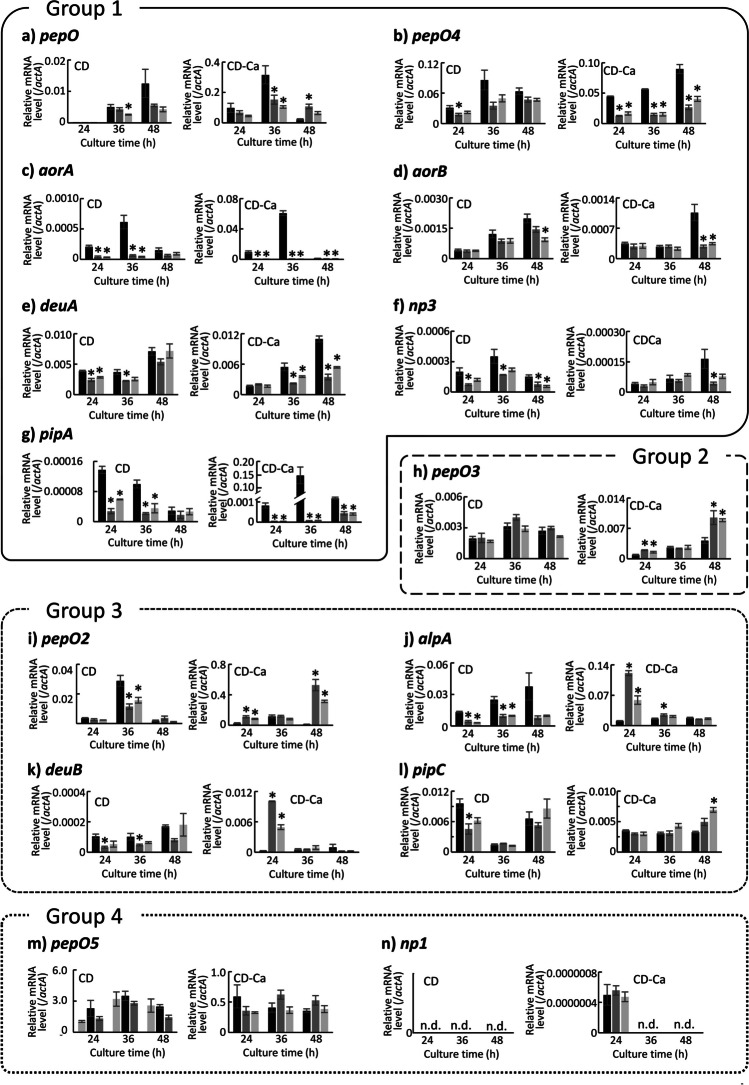
Transcriptional analysis result for endopeptidase genes in the control and ΔprtR strains cultured in liquid medium. Control or ΔprtR strains were grown for 24, 36, and 48 h in CD liquid medium or CD-casein liquid medium containing 1% casein as solo nitrogen source. The *y*-axis represents the relative mRNA levels of the extracellular peptidase gene compared to that of *actA*. The bars represent the mean of biological replicates (*n* = 3), and error bars represent the standard error. The black bars show the results of the control strain, and the two gray bars show the results of the ΔprtR strain. Statistical analysis was performed using the Dunnett method. *p < 0.05

The change patterns in transcription of endopeptidase genes due to the deletion of *prtR* were divided into the following four groups (Fig. 4). Group 1 was genes whose transcription was significantly decreased or tended to decrease in both CD and CD-Ca media due to the deletion of *prtR*. To this group belonged *pepO*, *pepO4*, *aorA*, *aorB*, *deuA*, *np3*, and *pipA*, encoding APase (PepO), APase (PepO4), aorsin A, aorsin B, deuterolysin A, metalloendopeptidase NP3, and glutamic endopeptidase (PipA), respectively. Group 2 was genes whose transcription was increased in CD-Ca medium and was hardly changed in CD medium by the deletion of *prtR*. This was the case for *pepO3*, encoding APase (PepO3). Group 3 was genes whose transcription was decreased or tended to decrease in CD medium but increased in CD-Ca medium in ΔprtR strain. To this group belonged *pepO2*, *alpA*, *deuB*, and *pipC*, encoding APase (PepO2), oryzin, deuterolysin B, and glutamic endopeptidase (PipC), respectively. Group 4 consisted of genes that were not altered by the deletion of *prtR*. To this group belonged *pepO5* and *np1* encoding APase (PepO5) and metalloendopeptidase NP1, respectively. However, the transcript level of *np1* was significantly low.

**Fig. 5:**
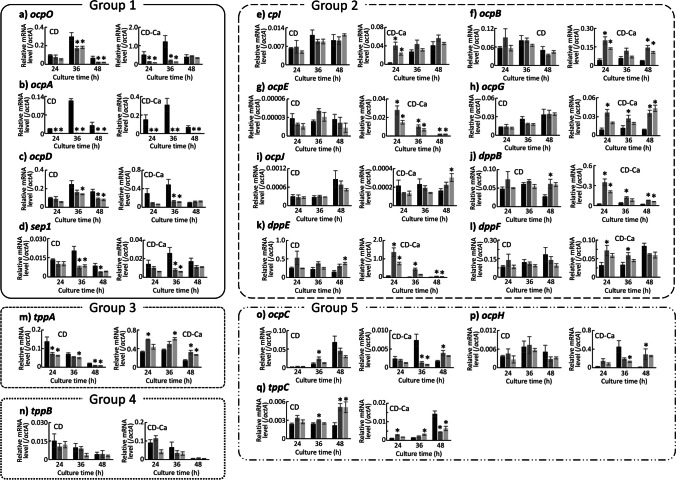
Transcriptional analysis result for exopeptidase genes in the control and ΔprtR strains cultured in liquid medium. Control or ΔprtR strains were grown for 24, 36, and 48 h in CD liquid medium or CD-casein liquid medium containing 1% casein as solo nitrogen source. The *y*-axis represents the relative mRNA levels of the extracellular peptidase gene compared to that of *actA*. The bars represent the mean of biological replicates (*n* = 3), and error bars represent the standard error. The black bars show the results of the control strain, and the two gray bars show the results of the ΔprtR strain. Statistical analysis was performed using the Dunnett method. *p < 0.05

For exopeptidase genes, the patterns of transcriptional changes due to the deletion of *prtR* could be divided into a total of five groups. Exopeptidase genes belonging to group 1 to 4 showed the same behavior as endopeptidase genes belonging to the same group due to the deletion of *prtR*, while the genes classified in the fifth group showed different behavior depending on the culture time in each medium (Fig. 5). The group 1 consisted of *ocpO*, *ocpA*, *ocpD*, and *sep1*, encoding CPases. Surprisingly, the transcription of *ocpA* was almost completely lost in the ΔprtR strain in both media at all cultivation times. The group 2 consisted of *cpI*, *ocpB*, *ocpE*, *ocpG*, and *ocpJ*, encoding CPases. In addition, the dipeptidyl peptidases *dppB*, *dppE*, and *dppF* were also classified into the group 2. The group 3 consisted of *tppA*, encoding tripeptidyl peptidase (TppA). The group 4 consisted of *tppB*, encoding tripeptidyl peptidase (TppB). The group 5 consisted of *ocpC*, *ocpH*, and *tppC*, encoding CPase (OcpC), CPase (OcpH), and tripeptidyl peptidase (TppC), respectively. Thus, dipeptidyl peptidase genes were classified in the same group, but tripeptidyl peptidase genes were classified in different groups.

### Transcription of peptidase genes in the ΔprtR strain cultured on solid medium

Solid-state cultures are known to be more productive and produce a greater variety of enzymes than liquid cultures (Murakami 1986). Therefore, to understand the role of PrtR in solid-state cultures, we analyzed the transcriptional levels of peptidase genes in solid-state cultures. Wheat bran was selected as a substrate for solid-state culture, as wheat bran culture can produce *A. oryzae* and *Aspergillus terreus* high extracellular proteolytic enzyme (Zanutto-Elgui et al. 2019; Abu-Tahon et al. 2020). In addition, wheat bran is available at a low cost as an agricultural residue, and its use to produce proteolytic enzymes as a culture substrate can contribute to resource recycling.

The ΔprtR and control strains were cultured for 24, 36, 42, and 48 h in wheat bran medium. The growth of both strains was similar (Supplemental Fig. S2A and B).

**Fig. 6:**
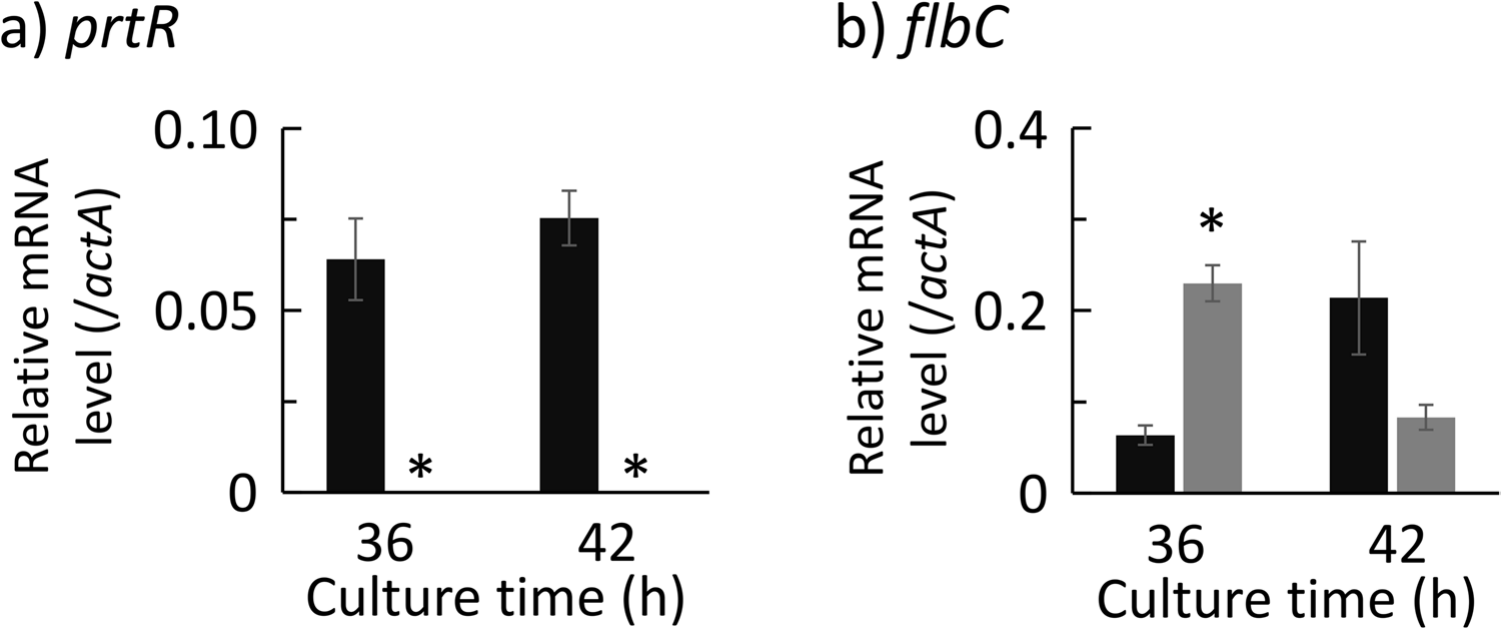
Transcriptional analysis result for transcription factor genes in the control and ΔprtR strains cultured on what bran medium. Control or ΔprtR strains were grown for 36 and 42 h in wheat bran medium. The *y*-axis represents the relative mRNA levels of the transcription factor gene compared to that of *actA*. The bars represent the mean of biological replicates (*n* = 3), and error bars represent the standard error. The black bars show the results of the control strain, and the gray bars show the results of the ΔprtR strain. Statistical analysis was performed using the *t* test method. *p < 0.05

The transcriptional levels of the genes encoding transcription factors, PrtR and FlbC, at 36 and 42 h are shown in Fig. 6. The *prtR* gene was transcribed at both cultivation times in the control strain but was not observed at 36 and 42 h in the ΔprtR strain as described above. Tanaka et al. (2016) have reported that *pepO* transcription is regulated by FlbC in a solid-state specific manner. To investigate the relationship between PrtR and FlbC in solid-state cultures, we analyzed the transcription levels of *flbC*. In the ΔprtR strain, the transcription of *flbC* was higher at 36 h and lower at 42 h than that in the control strain.

**Fig. 7:**
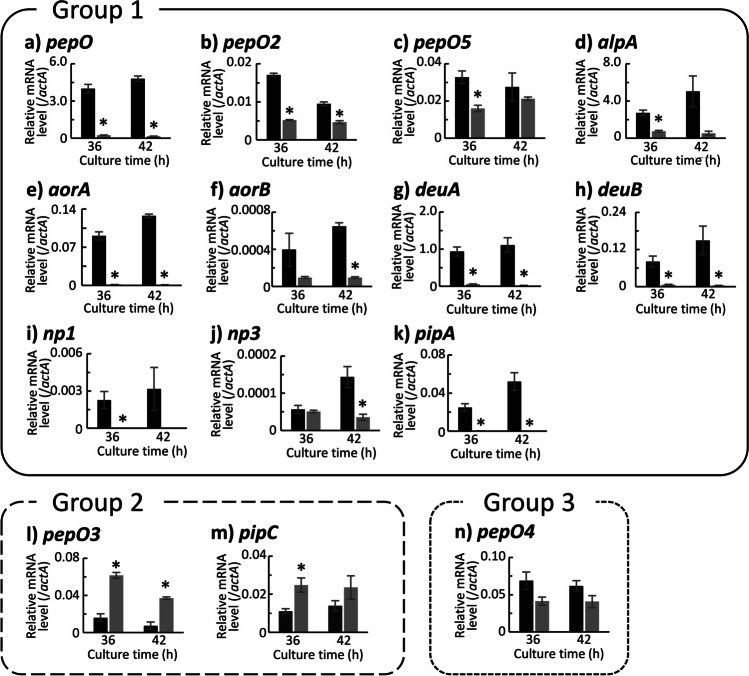
Transcriptional analysis result for endopeptidase genes in the control and ΔprtR strains cultured on what bran medium. Control or ΔprtR strains were grown for 36 and 42 h in wheat bran medium. The *y*-axis represents the relative mRNA levels of the extracellular peptidase gene compared to that of *actA*. The bars represent the mean of biological replicates (*n* = 3), and error bars represent the standard error. The black bars show the results of the control strain, and the gray bars show the results of the ΔprtR strain. Statistical analysis was performed using the *t* test method. *p < 0.05

The change patterns in transcription of endopeptidase genes due to the deletion of *prtR* in solid culture were divided into the following three groups (Fig. 7). Group 1 was genes whose transcription was decreased at both or either culture times due to the deletion of *prtR*. This group consisted of *pepO*, *pepO2*, *pepO5*, *alpA*, *aorA*, *aorB*, *deuA*, *deuB*, *np1*, *np3,* and *pipA*. Group 2 was genes whose transcription was increased at both or either culture times. This was the case for *pepO3* and *pipC*. Group 3 was genes with no transcriptional change at both culture times. The group 3 consisted of only *pepO4*.

**Fig. 8:**
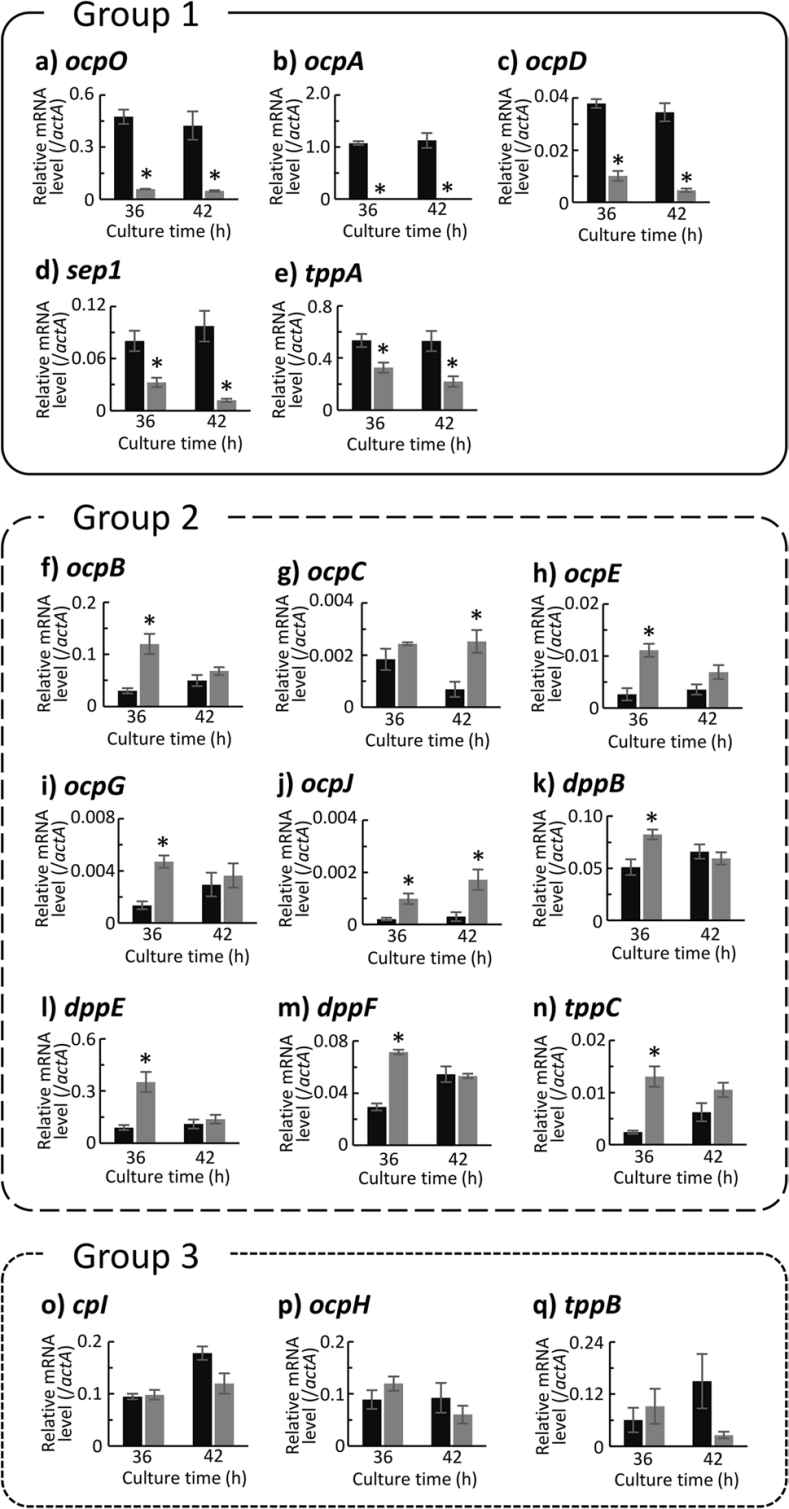
Transcriptional analysis result for exopeptidase genes in the control and ΔprtR strains cultured on what bran medium. Control or ΔprtR strains were grown for 36 and 42 h in wheat bran medium. The *y*-axis represents the relative mRNA levels of the extracellular peptidase gene compared to that of *actA*. The bars represent the mean of biological replicates (*n* = 3), and error bars represent the standard error. The black bars show the results of the control strain, and the gray bars show the results of the ΔprtR strain. Statistical analysis was performed using the *t* test method. *p < 0.05

For exopeptidase genes, the patterns of transcriptional changes due to the deletion of *prtR* could be divided into the above three groups (Fig. 8). The group 1 consisted of *ocpO*, *ocpA*, *ocpD*, *sep1*, and *tppA*. Transcript levels of all genes were significantly decreased at both incubation times. The group 2 consisted of *ocpB*, *ocpC*, *ocpE*, *ocpG*, *ocpJ*, *dppB*, *dppE*, *dppF*, and *tppC*. The group 3 consisted of *cpI*, *ocpH*, and *tppB*.

## Discussion

### PrtR is involved in promoting the production of peptidases in* A. oryzae*

*A. oryzae* PrtR is an orthologous protein of PrtT, which has been reported to positively regulate the transcription of several peptidase genes in *Aspergillus*. However, the detailed effect of PrtR on peptidase production has not yet been studied in *A. oryzae.* In this study, we aimed to identify the genes under the control of *A. oryzae* PrtR and elucidate their transcriptional regulatory mechanism. Therefore, we created a strain with the same genetic background as the wild-type strain, RIB40, but lacking the *prtR* gene. This is because many of the marker genes for transformation used in *A. oryzae* encode the proteins involved in nitrogen metabolism, and it was anticipated that amino acids or nucleic acids added to meet the requirements of these gene deletions and deficiencies would affect the transcription of proteolytic enzyme genes involved in nitrogen metabolism (Kitamoto 2002).

When the ΔprtR strain was grown on CD agar medium with a single protein other than soy protein as the nitrogen source, delayed growth and reduced density of conidia were observed (Fig. 1A). This was consistent with the results obtained for the *A. fumigatus* ΔprtT strain grown on CD/BSA agar medium. *A. fumigatus* PrtT positively regulates the transcription of several peptidase genes, and its deficiency causes a reduction in the transcription of peptidase genes (Bergmann et al. 2009). It was analogously presumed that *A. oryzae* PtrR is a positive transcriptional regulator of peptidase genes. Therefore, it was considered that the PrtR deletion should repress the transcription of the peptidase genes and reduce peptidase production. This could explain the delayed growth of the ΔprtR strain on plates containing BSA, gelatin, and casein as the single nitrogen source. However, the effect of PrtR deficiency was barely observed in the culture on CD/soy protein and CD/SM plates. As soy protein and skim milk are not pure proteins and contain some peptides and amino acids, even the ΔprtR strain can grow. Halo formation was not observed in the ΔprtR strain grown on CD/casein agar plates. This suggests that PrtR is strongly involved in the production of peptidases necessary for casein degradation (Fig. 1B). On CD/SM(− N) plates without NaNO_3_, the ΔprtR strain formed a smaller halo than the control strain (Fig. 1C). These results suggest that the ΔprtR strain could grow in the absence of inorganic nitrogen by inducing the transcription of peptidase genes that are not under the control of PrtR or that are under the control of PrtR but induced by other pathways. In contrast, on the CD/SM(+ N) agar plate, the ΔprtR strain did not form a halo, but the growth was not different from that of the control strain. These results indicate that inorganic nitrogen inhibits the production of peptidases encoded by genes that are not under the control of PrtR or induced by other pathways. This suggests that, in the presence of nitrate ions, the ΔprtR strain prefers to take up nitrate as a nitrogen source, rather than secreting peptidases to degrade surrounding proteins and taking up amino acids and oligopeptides as nitrogen sources. In contrast, in the control strain, no significant difference was noted in halo formation with or without sodium nitrate, but the colony diameter on the CD/SM(+ N) plate at day 5 was larger than that on the CD/SM(− N) plate. This indicates that the control strain utilized inorganic nitrogen even in the presence of PrtR. It was assumed that *A. oryzae* would utilize proteins and inorganic nitrogen in parallel in order to grow efficiently. The presence of inorganic nitrogen did not inhibit protein utilization.

In the *A. oryzae* genome, the *prtR* gene is located close to the starch-degrading enzyme gene cluster. The distance between the *prtR* gene and the closest *amyA* gene was only approximately 1 kbp. It was assumed that *prtR* is involved in the transcription of starch degradation-related gene clusters. However, no differences in colony diameter or growth were observed between the control strain and the ΔprtR strain on CD plates containing soluble starch as a single carbon source (Fig. 1A). Thus, PrtR was indicated not to be involved in the production of starch-degrading enzymes such as amylase.

### Culture conditions alter *prtR* transcript levels and the effects of its translation product

RT-qPCR was performed to determine which peptidase genes are regulated by PrtR. When the control strain was grown in CD-Ca liquid medium, *prtR* transcription increased compared to that in CD liquid medium, indicating that PrtR transcription was enhanced by proteins as the single nitrogen source. *A. oryzae* is likely to increase peptidase production by increasing the amount of PrtR via enhanced *prtR* transcription. Focusing on the overall transcription of peptidase genes, the number of peptidase genes whose transcriptional levels were altered by the deletion of PrtR was small in the CD liquid culture compared to that in the CD-Ca liquid culture. It would indicate that the enhancing effect of PrtR on transcription was low when inorganic nitrogen was the sole nitrogen source. *A. oryzae* might preferentially utilize inorganic nitrogen in the liquid culture and not need to secrete many peptidases.

The transcriptional levels of *prtR* in the control strain were similar in both wheat bran medium and CD liquid medium. However, the overall transcription of peptidase genes was higher in wheat bran culture. The number of peptidase genes altered by the deletion of *prtR* was higher in wheat bran culture than in CD liquid culture. In solid cultures, the effect of *prtR* regulation is extremely high, and the signal may be used for transcription promotion more effectively than in liquid cultures. Furthermore, there were some genes for which the mode of transcriptional regulation by PrtR changed from positive to negative or negative to positive in solid and liquid cultures. Glucose starvation in wheat bran medium leads to the release of carbon catabolite repression (CCR; Maeda et al. 2004). Thus, it is possible that genes under the control of PrtR and regulated by CCR have different modes of transcriptional regulation by PrtR depending on the culture conditions. Moreover, transcriptional regulation may differ from that in glucose-rich liquid cultures. Alternatively, signals other than nitrogen sources, such as water activity and temperature, may be important for the activation of peptidase gene transcription by PrtR in solid culture. Furthermore, other transcription factors, such as FlbC, may be closely related to PrtR. FlbC promotes the transcription of *pepO* and *deuA* in solid culture. The transcription levels of the *flbC* gene in the ΔprtR strain cultured for 36 h in wheat bran medium were significantly higher than those in the control strain. Furthermore, the transcript levels of the *flbC* gene increased in the ΔprtR strain, but the transcription of many peptidase genes, including *pepO* and *deuA*, decreased. This suggests that FlbC promotes the transcription of *prtR* which in turn promotes the transcription of peptidase genes. PrtR cannot cause an increase in transcription of peptidase genes in the ΔprtR strain. Therefore, it was considered that the feedback reaction would increase the transcription of the *flbC* gene, but the transcription of the peptidase genes in the ΔprtR strain would be reduced compared to the control strain.

### Proteolytic activities in culture broth are strongly suppressed by deletion of PrtR

The results of the peptidase activity assay and the transcriptional analysis were shown to be consistent with each other. Deletion of PrtR reduced acidic endopeptidase activity by about 84%. It would be due to mainly reflecting the decreased transcription of *pepO*, an endopeptidase that primarily works under acidic conditions (Takeuchi et al. 1995). Similarly, the transcription of *ocpO*, a representative CPase that works under acidic conditions (Morita et al. 2011), was decreased by the deletion of *prtR*. As a result, CPase activity is also reduced by 86%.

It was shown that the acidic endopeptidase activity of the control strain was inhibited to 21% and 34% by antipain and pepstatin A, respectively. Antipain is known as an inhibitor for cysteine peptidases and serine peptidases (Hoebeke et al. 1994), and pepstatin A inhibits APase (Purushothaman et al. 2019). It was presumed that antipain-sensitive acid endopeptidases in *A. oryzae* would be aorsins (AorA and AorB; Lee et al. 2003). The activity of the ΔprtR strain was not inhibited by antipain; therefore, PrtR would regulate the aorsin genes. This was consistent with the results of transcription analysis. In addition, 15% of the remaining activity in the control strain was completely eliminated by pepstatin A. Based on this result and the fact that transcription of APase genes occurred in the ΔprtR strain, even at a low level, it was suggested that these enzymes would be responsible for the residual acidic endopeptidase activity in the ΔprtR strain.

### PrtR can positively or negatively regulate the transcription of peptidase genes and the regulation altered by nitrogen source in the medium

Among the peptidase genes in liquid culture, the *ocpA* and *aorA* genes scarcely showed transcription in ΔprtR strain at any time point in CD-Ca medium. PrtR was considered essential for these transcriptions when the nitrogen source was a protein. Notably, the transcription of these genes was induced by PrtR even when inorganic nitrogen was the single nitrogen source. Although not at all time points, it was also shown that *deuA*, *pipA*, *ocpO*, *ocpD*, and *sep1* were positively regulated by PrtR in both liquid cultures containing inorganic and organic nitrogen. These results indicate that these genes were also transcriptionally promoted by PrtR, regardless of the type of nitrogen source. Some genes, such as *pepO*, encoding a typical APase, were positively regulated by PrtR when the nitrogen source was protein in the liquid medium. As PepO is a typical endopeptidase that works under acidic conditions (Takeuchi et al. 1995), this result is consistent with the significant reduction in proteolytic enzyme activity under acidic conditions in the ΔprtR strain. However, because some genes were negatively regulated by PrtR even when the protein was the single nitrogen source, it was suggested that PrtR selectively regulates peptidases to efficiently degrade proteins. Furthermore, certain genes were found to be transcriptionally promoted by PrtR when inorganic nitrogen was the sole nitrogen source, and their transcription was repressed when the protein was the single nitrogen source, while certain other genes showed the opposite behavior, which is unlikely to be reasonable in terms of nitrogen source utilization. The peptidase encoded by such genes may be used as a sensor to search for nitrogen sources in liquid culture. The fact that these peptidases were under the positive control of PrtR in wheat bran culture suggests that their roles may differ between liquid and solid cultures. In wheat bran medium, the transcription of genes encoding PepO (Kitano et al. 2002), AorA (Lee et al. 2003), DeuA (Tatsumi et al. 1991), and OcpA (Takeuchi et al. 1982) specifically produced in solid culture, was significantly reduced by the deletion of PrtR. This indicates that PrtR strongly and positively regulates transcription in solid culture.

Comparing the genes regulated by PrtR in liquid and solid cultures, the peptidase genes that were positively regulated in both cultures were *pepO*, *aorA*, *aorB*, *deuA*, *np3*, *pipA*, *ocpO*, *ocpA*, *ocpD*, and *sep1*. In particular, *ocpA* and *aorA* were significantly downregulated in the ΔprtR strain at all time points in both liquid and solid cultures. These results suggest that *ocpA* and *aorA* are strongly and positively regulated by PrtR in both liquid and solid cultures and that PrtR is essential for their transcription. Moreover, *pepO* and *pipA* were strongly downregulated in solid culture due to PrtR deficiency, whereas a certain level of transcription was observed in the ΔprtR strain in liquid culture. This suggests that transcription of these genes may be promoted by factors other than PrtR in liquid culture. In addition, there was another group, *deuA* and *deuB*, whose transcriptional levels were low in liquid culture but high and strongly promoted by PrtR in solid culture. These results also indicate that PrtR regulation is highly effective in solid culture. The *alpA* gene encoding oryzin, a typical alkaline protease (Nakadai et al. 1973), was positively regulated by PrtR in solid and CD liquid cultures but negatively regulated in CD-Ca liquid culture in the early stage of culture. However, *alpA* was not regulated by PrtR under any culture conditions during the late culture stages. These results suggest that *alpA* is regulated by nitrogen source and culture time through PrtR, as well as other factors. Transcription factor PacC promotes the transcription of alkaline peptidase genes under the alkaline condition (Tilburn et al. 1995), and the pH of the culture broth gradually increases in the late stage of culture when *A. oryzae* is cultured. This suggests that the transcription of the *alpA* gene in the early stage of *A. oryzae* culture would be controlled by PrtR, but the transcription would be controlled by PacC in the late stage of culture due to an increase in pH. Furthermore, PrtR and PacC would regulate the transcription of *alpA* through separate pathways, as the transcription of *prtR* did not show a noticeable decrease even at the late culture stage. Genes such as *pepO2*, *pipC*, *ocpC*, *tppA*, and *tppC* were positively or negatively regulated by PrtR depending on the culture conditions, and genes such as *pepO3*, *ocpB*, *ocpE*, *ocpG*, *ocpJ*, *dppB*, *dppE*, and *dppF* were upregulated by PrtR deficiency. Thus, PrtR may be a transcription factor that promotes and represses transcription. It is possible that the direction of PrtR regulation changed with culture conditions, either because PrtR regulation was altered by culture conditions through some signals, or because other transcription factors were activated to compensate for the reduced peptidase production caused by PrtR deficiency. Some genes are always negatively regulated by PrtR, but it could not be determined whether PrtR binds directly upstream of these peptidase genes and represses their transcription.

### PrtR’s control pattern is not exactly the same as PrtTs

Moreover, *pep1*, *alp1*, and Afu2g17330 are positively controlled by PrtT in *A. fumigatus* (Bergmann et al. 2009; Sharon et al. 2009). These peptidase genes were orthologs of *pepO*, *alpA*, and *sep1*, respectively, and these three genes were under the positive control of PrtR in *A. oryzae*. This indicated that the mode of PrtT regulation is conserved among closely related species (Sharon et al. 2009). However, Bergmann et al. (2009) reported that when the *A. fumigatus* ΔprtT strain was grown on a medium containing BSA as a nitrogen source, the transcription of *dppIV* was reduced and that of *dppV* was unchanged compared to the wild-type strain. A similar study conducted by Sharon et al. (2009) also reported a decrease in *dppIV* transcription in a medium with skim milk as the nitrogen source. In this study, the results for *dppB* (an ortholog of *A. fumigatus dppIV*) and *dppE* (an ortholog of Af*dppV*) differed from those of previous studies. This might be due to the difference in the protein used as a nitrogen source, but it is also possible that the regulatory mode of PrtR/PrtT for DppIV and DppV differs among species. Further experiments using BSA or skim milk as a nitrogen source will help elucidate the regulatory mechanism of dipeptidyl peptidase genes in *Aspergillus*.

The putative binding sequence 5′-CCGHCGG-3′ (H;A/C/T) of *A. niger* PrtT (Huang et al. 2020) or 5′-CHGH(D)CGG-3′ (H; A/C/T, D; A/G/T) of *P. oxalicum* PrtT (Chen et al. 2014) was not necessarily located within 1000 bp of the upstream region of all genes classified as the group 1. Therefore, the DNA-binding sequence of PrtR in *A. oryzae* may be different from that in *A. niger* or *P. oxalicum*. Furthermore, the DNA binding sequence of *A. niger* PrtT is derived from MEME analysis, not EMSA or DNA footprinting analysis. It may be necessary to confirm that PrtT binds this sequence. MEME analysis was performed on all genes in the group 1 or on all genes that showed changes in their transcript levels in the ΔprtR strain. However, each analysis did not reveal any statistically significant motifs common to all of the genes. MEME analysis has difficulty detecting DNA binding sequences that contain insertions/deletions. If PrtR’s binding sequence contained insertions/deletions like that of *P. oxalicum,* conserved sequence would not be found. Otherwise, these results of MEME analysis would suggest that some peptidase genes are directly regulated by PrtR, while others are indirectly regulated. In cases where PrtR promotes transcription, PrtR might bind to DNA alone or in a complex with other factors. Binding sequences may be different when binding alone and when binding as a complex. If transcription appears to be repressed by PrtR, it might be indirectly regulated by factors whose expression is promoted by PrtR. It was thought that genes regulated in different ways might be classified in the same group, since they were only evaluated at the final transcript level. To determine the direct binding sequence of PrtR, we are going to perform EMSAs with purified PrtR. This would clarify the differences between PrtR and orthologs of other organisms.

This study revealed that PrtR transcriptionally regulates peptidase genes and that the target genes under control are independent of substrate specificity. PrtR is involved in the regulation of most extracellular peptidase genes, suggesting that PrtR selectively regulates transcription to produce optimal peptidases depending on the culture conditions. Further studies are needed to elucidate the signals to which PrtR responds, the activation mechanism, and the DNA-binding sequence. Elucidation of the PrtR response network to the environment will provide important information on the peptidase production mechanism of *A. oryzae*, which has not yet been studied and will lead to the enhancement of the value of *A. oryzae* in the enzyme industry.

## Supplementary Information

Below is the link to the electronic supplementary material.

Supplementary file1 (PDF 936 KB)

## Data Availability

All data generated or analyzed during this study are included in this published article [and its supplementary information files].
