# Supplementary material for: Aspergillus oryzae PrtR alters transcription of individual peptidase genes in response to the growth environment
Source: Appl Microbiol Biotechnol. 2024 Jan 10;108:90. doi: 10.1007/s00253-023-12833-5 (PMC10781853; doi:10.1007/s00253-023-12833-5)
Supplement: Supplementary file 1 [file 253_2023_12833_MOESM1_ESM.pdf]

Supplemental Material

Journal name:
Applied Microbiology and Biotechnology

Manuscript Title:
Aspergillus oryzae PrtR alters transcription of individual peptidase genes in response to the growth environment

The name(s) of the author(s):
Rika Numazawa[1], Yukako Tanaka[2], Sawako Nishioka[2], Ryotaro Tsuji[2], Hiroshi Maeda[2], Mizuki Tanaka[1, 2], Youhei Yamagata[1,2]*

The affiliation(s) and address(es) of the author(s):
[1] Department of Applied Biological Science, United Graduate School of Agricultural Science, Tokyo University of Agriculture and Technology, 3-5-8 Saiwai-cho, Fuchu, Tokyo 1838509, Japan
[2] Department of Applied Biological Chemistry, Graduate School of Agriculture, Tokyo University of Agriculture and Technology, 3-5-8 Saiwai-cho, Fuchu, Tokyo 1838509, Japan

The e-mail address, telephone and fax numbers of the corresponding author:
E-mail: y-yama@cc.tuat.ac.jp; Phone: +81 423675706; Fax: 81 423675706

Table S1 Nucleotide sequences of primers used in this study.

Primer name	Sequence (5' to 3')
For deletion mutant construction	
prtR_5'_F	TGATAAGAATGGCGATACTGCAGACTAC
prtR_3'_R	CAACTTGGCTGATCGATGATACTTACG
prtR_3'_F	GAATAATCGGCGAGTAGAACAAGCTG
prtR_5'_R	GTATATCCTATATTCAATCACGCGCCAGAC
creloxp_F	ATCGCTGGATCAACGCTGGGATCAC
creloxp_R	TGTAAAACGACGGCCAGTGCCAAGC
pyrG_Fw	GGTAATGTGCCCCAGGCTTGTCAG
pyrG_Rv	GGCCCGAGAGGACTATTCCGAG
creloxp_5'_F	CCTCATATGGAAGAACGTCAGACGTGAC
creloxp_3'_R	GGTACCCCTCGAAACATAACTTCGTATA
prtR_up2049_F	CATCCGGGCCGCGAATGATATGATATC
prtR_up967_R	GCATAGTGGCATGATAGCGTTCTGCC
prtR_down97_F	GGATATACGATATGTCTCCCCGTAC
prtR_down1045_R	GGGTGGAGAGTATATGATGGTACTGC
ligD_up_F	GAGAAGCCCTGTCGCACTTTATCGG
ligD_up_R	CAATCACACGCGCTATTACGCCAC
ligD_down_F	TTAAGGAGTCCGCAGCTGAAGATGG
ligD_down_R	GCCTAGAAGGGTTCATTGTGCTGC
For quantitative RT-qPCR	
prtR-RT-F	GTTCTCAATGCTCGTTCGGA
prtR-RT-R	GAAATGAGTGTGGCATGACCTC
actA-RT-F	TCATGAAGTGTGATGTTGATGTCC
actA-RT-R	GGCAAGGGCGGTGATTTC
pepO-RT-F	CTCCTATGACTTCGGCTTCATC
pepO-RT-R	GCCCTTTCCGACAGAGTAACC
pepO2-RT-F	CGATGGTAGTGGGTCTGGTG
pepO2-RT-R	CCCGCTGTTGAAAGGATTG
pepO3-RT-F	CGATCACGACAAATACCAGG
pepO3-RT-R	ATCTCCATCCGCAACAGC
pepO4-RT-F	GAATACTAGAGACGACCGAGAAC
pepO4-RT-R	CACAATAACGCACAGCATCC

Table S1 Nucleotide sequences of primers used in this study (continued).

Primer name	Sequence (5' to 3')
pepO5-RT-F	CGACACCGGAAACGAATTCTC
pepO5-RT-R	CCCACAACCACACCAAACAC
alpA-RT-F	CTTTCTGCCTGGATTGGCTC
alpA-RT-R	CAACTCCTTGATGCGCTTGG
aorA-RT-F	TTCGGGTGGTGGCTTCAG
aorA-RT-R	ATGGATAGGGAGGGTTGTGG
aorB-RT-F	CGAAGAACAAGCAACGACCAG
aorB-RT-R	CGCTTTCGTAGTAGGGATAGG
deuA-RT-F	CGCCAACTGCGACATCTAC
deuA-RT-R	CATAGCCGTAGCCCAAGTCC
deuB-RT-F	CGCCAACTGCGACATCTAC
deuB-RT-R	CATAGCCGTAGCCCAAGTCC
np1-RT-F	CATCCGTGCTTACCCATTCTC
np1-RT-R	CCTTGAACTCGGGCTTTG
np3-RT-F	ACGGAATGGGAATGTGGGTTT
np3-RT-R	CAGGGATGTGTAGTTCAGTGGGT
pipA-RT-F	ATCTCCATCCGCAACAGC
pipA-RT-R	AGCAGGCGTGACCAACAAG
pipC-RT-F	AAAGGAACGAGCGACAAGAAAG
pipC-RT-R	AGACTGTCACTGCTCCTGAG
ocpO-RT-F	CTACTTCAACCGCACTGACG
ocpO-RT-R	GTTCGTCCGCTCAATCACAC
cpI-RT-F	AGTCCCATACTACCAGCCCATC
cpI -RT-R	GCCGTTCCATTCGTCTTGTAGC
ocpA-RT-F	GAAGCAGGTAGTCAGAAAGGTG
ocpA-RT-R	CGATGAGTTGTCTGTAGGTGTTG
ocpB-RT-F	GAGCAGAACAACACATTTTACG
ocpB-RT-R	CGTTTTCGGTGGTATTGGC
ocpC-RT-F	TTCCACAAGCACCATCACTC
ocpC-RT-R	CAACACCCTCGCTCCATTC
ocpD-RT-F	GACGAGTGGACCGAAGGATAC
ocpD-RT-R	AACATTCCCAGCCACAACCC

Table S1 Nucleotide sequences of primers used in this study (continued).

Primer name	Sequence (5' to 3')
ocpE-RT-F	AGGAAATCTCCAAGGTCGCC
ocpE-RT-R	CGTGTCCAGCCTCCCAAAC
ocpG-RT-F	ACGGCTTTATTGGCCATCTC
ocpG-RT-R	TCTAGGACCGAGGGTTCGTG
ocpH-RT-F	GCCCACAGCATTCAACTACC
ocpH-RT-R	AACAACCTGACCAAGTGACG
ocpJ-RT-F	GACTTATCATCTCTTTTGGGGC
ocpJ -RT-R	CAACAACATCACGCACATAAGC
sep1-RT-F	ATCCATCTCCAGTGATGAGGATTCG
sep1-RT-R	AATAACCCCATTCAGTGCAAACCTG
dppB-RT-F	CCTGTTTCTGACTGGCGTTTC
dppB-RT-R	CTCGTAGCCCTCCTCATTGGTC
dppE-RT-F	CAAACGGCACAGCATACAACGAAG
dppE-RT-R	CGGCATTGAATCGGGTGGTG
dppF-RT-F	CATGAGCTTGGTCGCCGATTC
dppF-RT-R	GCTCCTCAGTGCTCAACAGAG
tppA-RT-F	ATTGGCTTGATGGGTCTGCG
tppA-RT-R	AGTCTCGGTGCCGTCGTTAG
tppB-RT-F	ATGACCAACGACGGCACCAAC
tppB-RT-R	AGAAACCGCCCGAGGAGAAG
tppC-RT-F	CCAAATGGGAAGGGCTCTACAAC
tppC-RT-R	ACAATACCAGCAAACACAGGCG

Table S2 Peptidase genes whose transcription were analyzed in this study.

Types of peptidases		Gene name	AO number
Endo peptidases	Aspartic endopeptidases (APase)	*pepO*	AO090120000474
		pepO2	AO090012000667
		pepO3	AO090012000209
		pepO4	AO090009000228
		pepO5	AO090010000644
	Serine endopeptidases	*alpA*	AO090003001036
		aorA	AO090026000083
		aorB	AO090023000263
	Metallo endopeptidases	*deuA*	AO090010000493
		deuB	AO090001000135
		np1	AO090011000036
		np3	AO090138000160
	Glutamate endopeptidases	*pipA*	AO090023000393
		pipC	AO090010000100
Exo peptidases	Serine-type carboxypeptidases (CPase)	*ocpO*	AO090020000351
		cpI	AO090103000026
		ocpA	AO090012000706
		ocpB	AO090701000220
		ocpC	AO090103000153
		ocpD	AO090023000382
		ocpE	AO090026000680
		ocpG	AO090120000232
		ocpH	AO090010000534
		ocpJ	AO090023000848
	Putative serine-type carboxypeptidases	*sepI*	AO090102000079
	Dipeptidyl-peptidases	*dppB*	AO090023000602
		dppE	AO070340000111
		dppF	AO090005000697
	Tripeptidyl-peptidases	*tppA*	AO090166000084
		tppB	AO090011000235
		tppC	AO090005001380

Supplemental Material

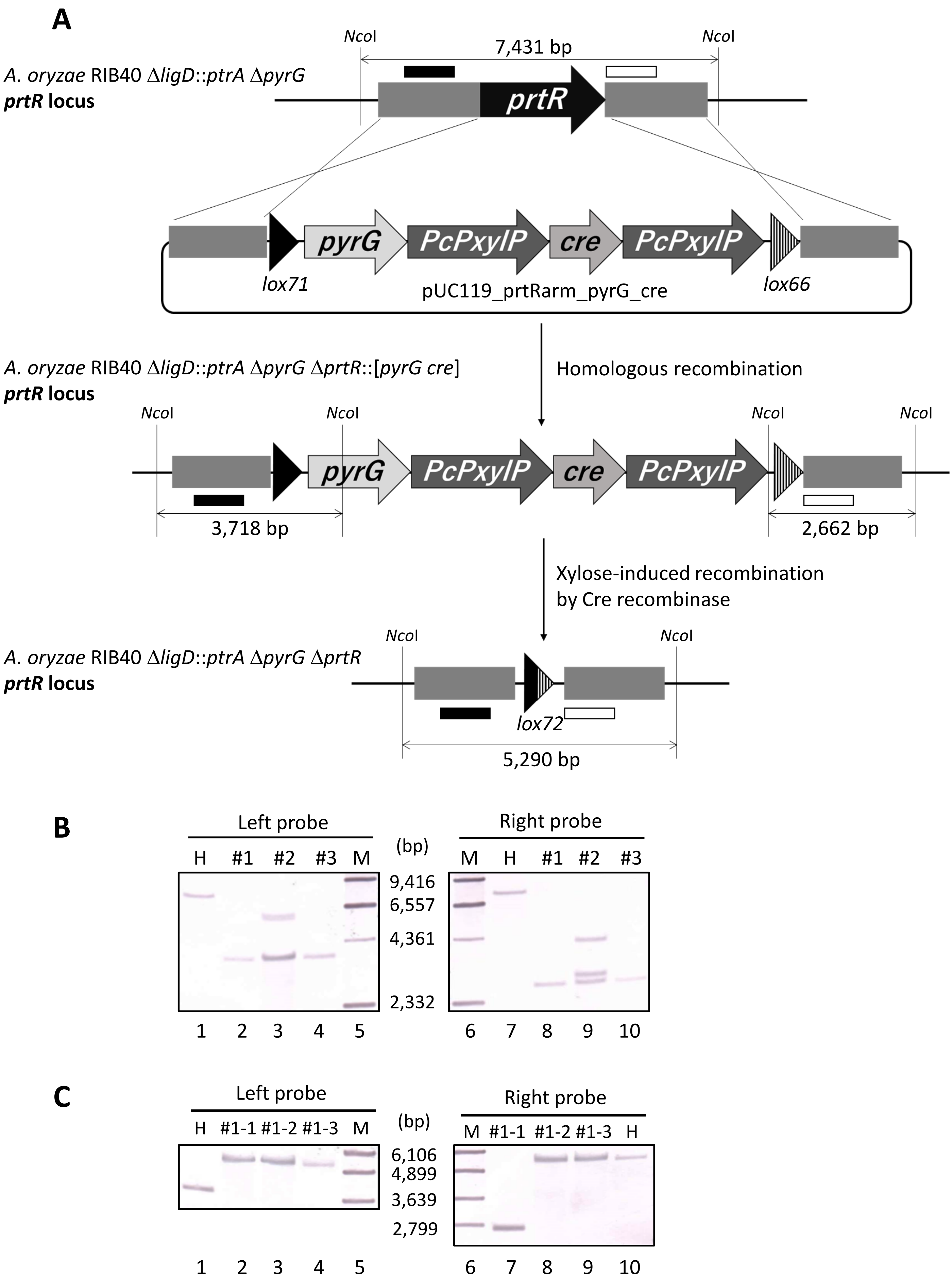

Fig. S1 (continued)

D

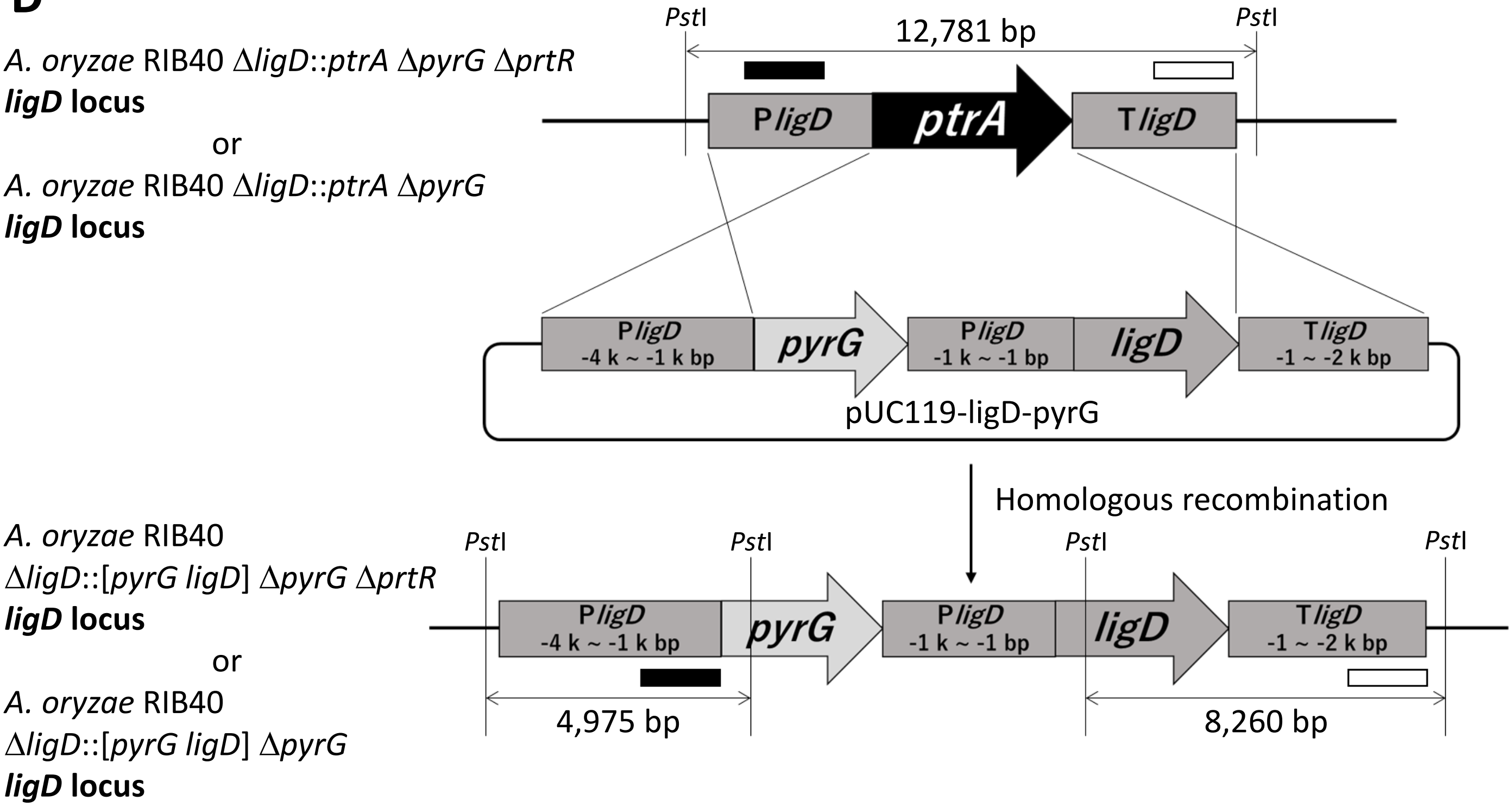

E

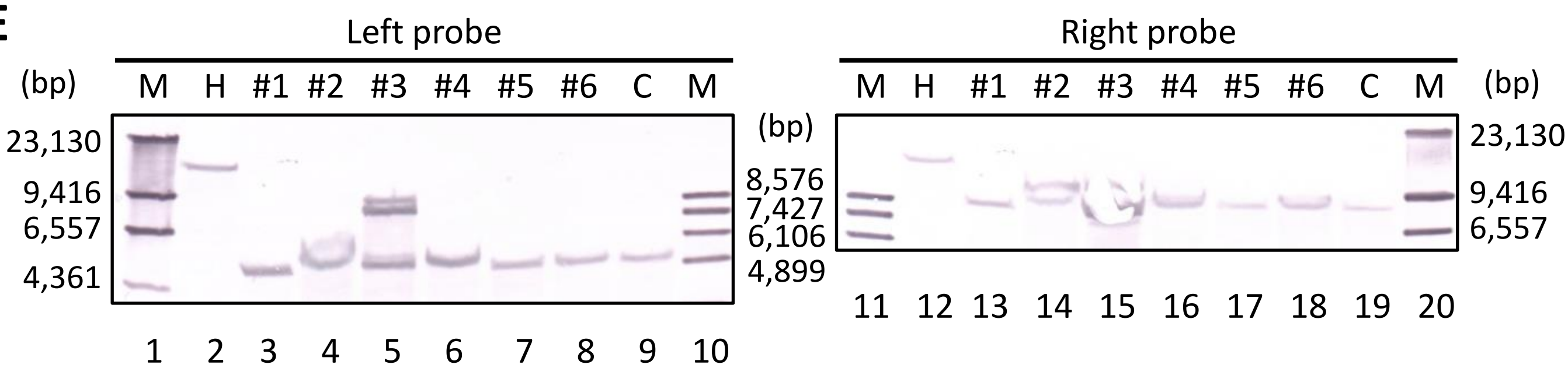

Fig. S1 (continued)

Fig. S1 Summary of ΔprtR construction and confirmation of the mutant strains by Southern blotting. (continued)

A) Schematic presentation of *prtR* gene deletion. The *prtR* gene of the host strain (Δ*ligD*::*ptrA* Δ*pyrG*) was replaced by a DNA fragment carrying the *pyrG* gene and the Cre-*loxP* system in pUC119_prtRarm_pyrG_cre. The obtained strain was designated as Δ*ligD*::*ptrA* Δ*pyrG* Δ*prtR*::[*pyrG cre*]. Then, the expression of Cre-*loxP* system was induced in the induction medium containing xylose, and the DNA fragment containing the *pyrG* gene was removed by loop-out. The resulting strain was named ΔligD ΔpyrG ΔprtR. The genomic DNAs of the mutant strains were digested with *Nco*I and the target DNA fragment was detected using two probes, the left probe (black box) and the right probe (open box), respectively.

B) Southern blot analysis of Δ*ligD*::*ptrA* Δ*pyrG* Δ*prtR*::[*pyrG cre*] candidate strains. The left and right panels indicate the result using left and right probe, respectively. Lane 1 and 7; host strain (Δ*ligD*::*ptrA* Δ*pyrG*), lane 2-4 and 8-10; candidate strains (#1-#3), lane 5 and 6; markers.

C) Southern blot analysis of the ΔligD ΔpyrG ΔprtR candidate strains. The left and right panels indicate the result using left and right probe, respectively. Lane 1 and 10; host strains (Δ*ligD*::*ptrA* Δ*pyrG* Δ*prtR*::[*pyrG cre*]), lane 2-4 and 7-9; candidate strains (#1-1-#1-3), lane 5 and 6; markers.

D) Schematic presentation of *ligD* and *pyrG* complementation to the ΔligD ΔpyrG ΔprtR strain. The *ptrA* gene in a *ligD* locus was replaced by a DNA fragment carrying the *pyrG* and *ligD* genes in pUC119-ligD-pyrG. The resulting strain was named ΔprtR. DNAs of the mutant strains were digested with *Pst*I and the target DNA fragment was detected using two probes, the left probe (black box) and the right probe (open box), respectively.

E) Southern blot analysis of the ΔprtR candidate strains. The left and right panels indicate the result using left and right probe, respectively. Lane 1, 10, 11, and 20; markers, lane 2, and 12; host strains (Δ*ligD*::*ptrA* Δ*pyrG* Δ*prtR*), lane 3-8 and 13-18; candidate strains (#1-#6), lane 9, and 19; control strains (Δ*ligD*::[*pyrG ligD*] Δ*pyrG*).

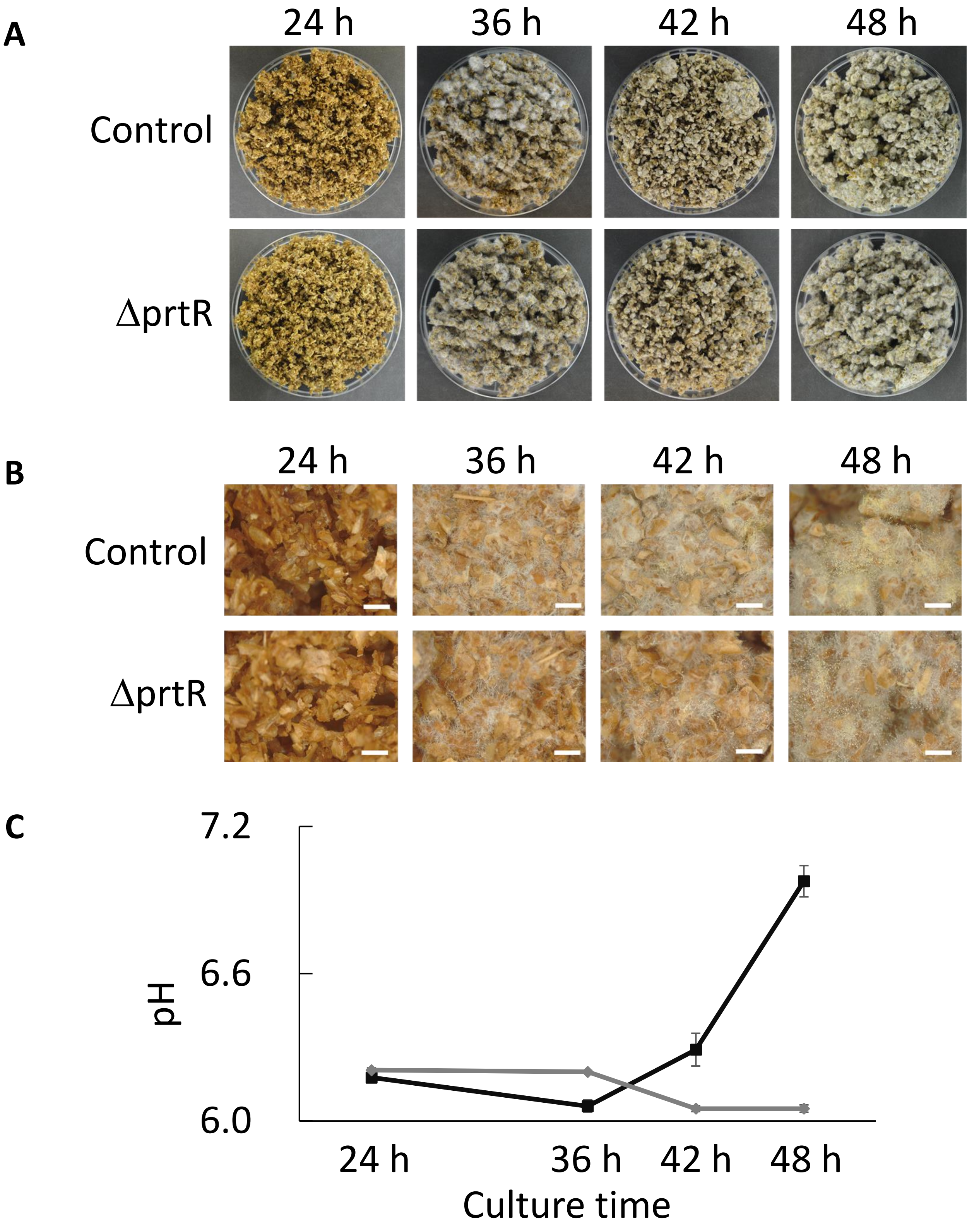

Fig. S2 The growth of the ΔprtR strain on wheat bran as a solid medium.

A) The growth of the ΔprtR strain and the control strain after inoculating 3 x 10^{7} of each conidia in 10.2 g of wheat bran and incubating at 30 ºC for 24, 36, 42, and 48 h.
B) Enlarged photograph of part of the result shown in A. Bars indicate 2 mm.
C) The pH of the culture medium of the ΔprtR strain and the control strain after cultivation on wheat bran. Ten milliiter of water was added to the culture, stirred well, and the pH of the filtrate through Miracloth was measured. Error bars indicate the standard error (n=3). The black bars show the results of the control strain and the gray bars show the results of the ΔprtR strain.
